# Neuroprotective effect of a novel Chinese herbal decoction on cultured neurons and cerebral ischemic rats

**DOI:** 10.1186/s12906-016-1417-1

**Published:** 2016-11-04

**Authors:** Fanny Chui-Fun Ip, Yu-Ming Zhao, Kim-Wan Chan, Elaine Yee-Ling Cheng, Estella Pui-Sze Tong, Oormila Chandrashekar, Guang-Miao Fu, Zhong-Zhen Zhao, Nancy Yuk-Yu Ip

**Affiliations:** 1 Division of Life Science, The Hong Kong University of Science and Technology, Clear Water Bay, Hong Kong, China; 2 State Key Laboratory of Molecular Neuroscience, The Hong Kong University of Science and Technology, Clear Water Bay, Hong Kong, China; 3 Guangdong Key Laboratory of Brain Science, Disease and Drug Development, HKUST Shenzhen Research Institute, Shenzhen, Guangdong China; 4 School of Chinese Medicine, Hong Kong Baptist University, Hong Kong, China; 5 Present Address: Department of Pharmacology, School of Basic Medical Sciences, Capital Medical University, Beijing, 100069 China

**Keywords:** TCM, Stroke, MCAO, Neuroprotection, Excitotoxicity, NMDA receptor, ERK, CREB

## Abstract

**Background:**

Historically, traditional Chinese medicine has been widely used to treat stroke. Based on the theory of Chinese medicine and the modern pharmacological knowledge of herbal medicines, we have designed a neuroprotective formula called Post-Stroke Rehabilitation (PSR), comprising seven herbs – *Astragalus membranaceus* (Fisch.) Bunge, *Salvia miltiorrhiza* Bunge, *Paeonia lactiflora* Pall., *Cassia obtusifolia* L., *Ligusticum chuanxiong* Hort., *Angelica sinensis* (Oliv.) Diels, and *Glycyrrhiza uralensis* Fisch. We aim to examine the neuroprotective activity of PSR in vitro and in vivo, and to explore the underlying molecular mechanisms, to better understand its therapeutic effect and to further optimize its efficacy.

**Methods:**

PSR extract or vehicle was applied to primary rat neurons to examine their survival effects against *N*-methyl-d-aspartate (NMDA)-elicited excitotoxicity. Whole-cell patch-clamp recording was conducted to examine the NMDA-induced current in the presence of PSR. ERK- and CREB-activation were revealed by western blot analysis. Furthermore, PSR was tested for CRE promoter activation in neurons transfected with a luciferase reporter. The protective effect of PSR was then studied in the rat middle cerebral artery occlusion (MCAO) model. MCAO rats were either treated with PSR extract or vehicle, and their neurobehavioral deficit and cerebral infarct were evaluated. Statistical differences were analyzed by ANOVA or t-test.

**Results:**

PSR prominently reduced the death of cultured neurons caused by NMDA excitotoxicity in a dose-dependent manner, indicating its neuroprotective property. Furthermore, PSR significantly reduced NMDA-evoked current reversibly and activated phosphorylation of ERK and CREB with distinct time courses, with the latter’s kinetics slower. PSR also triggered CRE-promoter activity as revealed by the increased expression of luciferase reporter in transfected neurons. PSR effectively reduced cerebral infarct and deficit in neurological behavior in MCAO rats when PSR decoction was administered starting either 6 days before or 6 h after onset of ischemia.

**Conclusions:**

PSR is neuroprotective both in vitro and in vivo – it protects cultured neurons against NMDA excitotoxicity, and effectively reduces ischemic injury and neurobehavioral deficit in MCAO rats in both the pre- and post-treatment regimens. The underlying neuroprotective mechanisms may involve inhibition of NMDA receptor current and activation of ERK and CREB. This study provides important preclinical data necessary for the further development of PSR for stroke treatment.

**Electronic supplementary material:**

The online version of this article (doi:10.1186/s12906-016-1417-1) contains supplementary material, which is available to authorized users.

## Background

Stroke, which can be hemorrhagic or ischemic, is caused by the bursting or blockade of blood vessels, respectively. It is one of the leading causes of death and disability worldwide, and 85 % of all stroke cases are ischemic [1–3]. Recombinant tissue plasminogen activator (rtPA), an enzyme that breaks down blood clot aiming to restore blood reperfusion, is the only FDA-approved pharmacological treatment for ischemic stroke [1–3]. However, its application is restricted because of a narrow administration time window of ~3 to 4.5 h after the initiation of ischemia [4]. Hence, there is an urgent need for novel stroke treatments, and different neuroprotective strategies that counteract the molecular cascades responsible for ischemic injury have been proposed [3, 5–8]. Mechanisms that contribute to ischemic injury include glutamate excitotoxicity, oxidative damage, inflammation and apoptosis; molecular targets for neuroprotection include calcium channel, glutamate receptor, GABA_A_ receptor, PSD95, nitric oxide synthase and extracellular adhesion molecule [3, 5–8]. In particular, one neuroprotective strategy targets the N-methyl-d-aspartate receptor (NMDAR) which plays a critical role in mediating glutamate excitotoxicity and neuronal death [9–11]. Interestingly, NMDAR also mediates pro-survival signaling that is beneficial to neuronal survival upon cerebral ischemic attack, via Akt and ERK activation [7]. Furthermore, NMDAR-mediated ERK activation in turn stimulates CREB, a transcriptional factor which regulates a number of genes critical for long-term neuroprotective effect [12]. A prevalent view has emerged to explain the two opposing effects – excitotoxicity and pro-survival – on neurons upon NMDAR activation: they are mediated by different NMDAR subtypes containing GluN2B and GluN2A, respectively [7].

Many neuroprotective compounds have been demonstrated to be effective in animal models of cerebral ischemia, and some candidates are being actively pursued in clinical trials (e.g. clinical trials with identifiers NCT02315443, NCT02222714, NCT01454154, NCT01221246, NCT01502761, NCT02144584 and NCT02535611), including four that mitigate the excitotoxicity mediated through NMDAR (NCT02315443, NCT01502761, NCT02144584 and NCT02535611), by blocking NMDAR channel current or channel interaction with PSD95 [6, 8, 11, 13, 14]. It is known that some traditional Chinese medicine (TCM) preparations exert neuroprotection with few or no adverse effects, and some can inhibit NMDAR current [15, 16].

According to traditional Chinese medicine, stroke is caused by *Qi* deficiency and blood stasis [17, 18]. For centuries, various multi-herbal formulas have been used by Chinese medicine practitioners to treat various diseases [19–21]. Based on the theory of TCM, together with modern pharmacological knowledge of herbal medicines, a neuroprotective formula called Post-Stroke Rehabilitation (PSR), comprising seven herbs (Table 1) has been designed. Most chosen herbs have been used in herbal mixtures for stroke treatment, including Buyang Huanwu, Sheng Yu, Xiaoxuming, Gualou Guizhi and Wen Dan Decoctions as well as BNG-1, Hua Tan Tong Luo formula and Nao-Shuan-Tong [22–30]. Moreover, extracts and/or isolates from all constituent herbs have been demonstrated to be neuroprotective in cell and/or animal models. For examples, extracts from *C. obtusifolia* and *G. uralensis* could protect cultured neurons against experimental induced-neurotoxicity and *P. lactiflora* extract could induce proliferation of injured neurons or cultured Schwann cells [31–35]; active molecules such as calycosin isolated from *A. membranaceus*, tanshinones from *S. miltiorrhiza*, chrysophanol from *C. obtusifolia*, and ferulic acid and ligustilide from *L. chuanxiong* and *A. sinensis* were neuroprotective in rodent ischemic models [36–41]. Four of the selected herbal components – *A. membranaceus*, *P. lactiflora*, *L. chuanxiong* and *A. sinensis* – are among the most commonly used herbs for stroke treatment [42]. In addition to the prominent neuroprotective properties of *S. miltiorrhiza*, *G. uralensis* and *C. obtusifolia* (Table 1), they were included in PSR because *S. miltiorrhiza*, can promote blood circulation and remove blood stasis [43]; *G. uralensis* can harmonize the actions of multiple herbs [43]; *C. obtusifolia* has been shown to reduce hyperlipidemia [44, 45], a known risk factor for stroke [45]. The amount of each herb used in the PSR mixture is in accordance with the recommendation stated in the Chinese Pharmacopoeia [43].

**Table 1:** Herbal components of PSR and their implications for stroke treatment

Component of PSR	Implications for stroke treatment^a^
1. *Astragalus membranaceus* (Fisch.) Bunge (Huangqi)	A component of Buyang Huanwu Decoction^b^ (BYHWD), Sheng Yu Decoction and BNG-1 [22, 23, 27]; used with Honghua to treat stroke patients [17]; *its isolates, calycosin and formononetin, were neuroprotective in cerebral ischemic rats* [40, 81]
2. *Salvia miltiorrhiza* Bunge (Danshen)	A component of Hua Tan Tong Luo formula and BNG-1 [27–29]; *polysaccharide extract and active isolates, lithospermic acid, salvianolic acid B, cryptotanshinone, tanshinone I and tanshinones IIA-B, could reduce damages caused by cerebral ischemia in rodents* [41, 82–85]
3. *Paeonia lactiflora* Pall. (Chishao)	A component of BYHWD [22] and Nao-Shuan-Tong [30]; *showed proliferative effects on injured rat peripheral neurons and Schwann cells* [31, 32]*; an isolate, paeoniflorin, was neuroprotective in cerebral ischemic rats* [86]
4. *Cassia obtusifolia* L. (Juemingzi)	Used for hyperlipidemia treatment [44]; *showed neuroprotective effects in hippocampal neurons and in cell and animal models of Parkinson’s disease in mice* [33, 34]*; an isolate, chrysophanol, showed neuroprotective effect in ischemic mice* [87]
5. *Ligusticum chuanxiong* Hort. (Chuanxiong)	A component of BYHW, Sheng Yu, and Xiaoxuming Decoctions [22–24]; *butylidenephthalide, ferulic acid and ligustilide isolated from Chaunxiong were neuroprotective in rodent cells and ischemic rats* [36–38, 88]
6. *Angelica sinensis* (Oliv.) Diels (Danggui)	A component of BYHWD and Sheng Yu Decoction [22, 23]; *ferulic acid and ligustilide isolated from Danggui showed neuroprotection in rodent cells and ischemic rats* [36–38]
7. *Glycyrrhiza uralensis* Fisch. (Gancao)	A component of BNG-1, Xiaoxuming, Gualou Guizhi, and Wen Dan Decoctions [24–27]; used to treat diabetic acute ischemic stroke [89]; *liquiritin, a major constituent, enhanced the neurotrophic effect of NGF* [90]*, showed protection against Aβ-induced toxicity in rat cortical neurons and ischemic mice* [35, 91]

^a^Examples and references shown are selections highlighted by the authors

^b^Buyang Huanwu, Sheng Yu, Xiaoxuming, Gualou Guizhi, and Wen Dan Decoctions, BNG-1, Hua Tan Tong Luo formula and Nao-Shuan-Tong are herbal mixtures that have been used to treat stroke patients in China

Neuroprotection data obtained from in vitro and animal studies are written in *italics*

To further understand if PSR is an effective treatment for stroke, preclinical and clinical studies are required. In this study, we aim to understand the action mechanism of PSR using in vitro primary embryonic neuron cultures, which express predominately GluN2B-containing NMDA receptors [46], and in vivo using the rat middle cerebral artery occlusion (MCAO) model. Our study indicates that PSR can protect against NMDA-induced excitotoxicity, inhibit NMDA-induced current and activate the pro-survival ERK/CREB signaling in cultured primary embryonic neurons. Furthermore, PSR is neuroprotective and attenuates neurological impairments in MCAO rats when administered either before or after the onset of ischemia. The present findings offer insights into the underlying multi-targeted neuroprotective mechanism of PSR and provide a scientific basis for its further pharmacological development for managing stroke and other neurological diseases.

## Methods

### Chemicals and reagents

Unless otherwise specified, all chemicals, antibodies, and culture reagents were purchased from Sigma (MO, USA), Cell Signaling Technology (MA, USA), and Life Technologies (CA, USA), respectively.

### Animals and the MCAO model

Sprague–Dawley (SD) rats were obtained and reared from the Animal and Plant Care Facility of The Hong Kong University of Science and Technology. Rats were provided with food and water ad libitum under a 12/12 h light/dark cycle. Transient focal cerebral ischemia was induced by intra-luminal MCAO as described previously [47, 48]. Male SD rats (280–320 g) were anesthetized and placed on heating pad to maintain rectal temperature at 37 °C. A nylon filament was advanced from the right external carotid artery into the lumen of the internal carotid artery to occlude the right MCA for 2 h, and the filament was subsequently withdrawn to allow reperfusion. The cerebral blood flow of the rats was monitored closely with Laser Doppler (PF 5010 – LDPM unit; PERIMED) during the operation. Sham-treated rats were subjected to the same operative procedure without the insertion of the nylon filament. The right femoral artery was cannulated to monitor arterial blood gases, pH, electrolytes, and hematocrit. All procedures were approved by the Animal Ethics Committee of The Hong Kong University of Science and Technology.

### Plant collection and identification

PSR comprises *A. membranaceus*, *S. miltiorrhiza*, *P. lactiflora*, *C. obtusifolia*, *L. chuanxiong*, *A. sinensis*, and *G. uralensis* (Table 1). All herbs were purchased from Lee Hoong Kee Limited, Hong Kong, and collected from different regions of China: *A. membranaceus* (roots; voucher ID: TCM208-4) and *G. uralensis* (roots and rhizomes; TCM080-4) from Inner Mongolia Autonomous Region; *S. miltiorrhiza* (roots and rhizomes; TCM012-4), *P. lactiflora* (roots; TCM037-3) and *L. chuanxiong* (rhizomes; TCM111-3) from Sichuan; *C. obtusifolia* (seeds; TCM238-2) from Anhui; *A. sinensis* (roots; TCM800-2) from Gansu. All herbs were authenticated by one of the authors (Dr. Guang-Miao Fu, a pharmacognosist) and a voucher specimen of each herb with identification number indicated above was deposited in the herbarium of the Biotechnology Research Institute at the Hong Kong University of Science and Technology.

### PSR preparation and HPLC fingerprinting

The PSR formula was prepared by the National Engineering Research Center for Modernization of Traditional Chinese Medicine, Zhuhai, China. The amount of each herb used in the mixture is in accordance with the recommendation stated in the Chinese Pharmacopoeia [43]. Briefly, mixture containing *A. membranaceus* (15 g), *S. miltiorrhiza* (10 g), *P. lactiflora* (7.5 g), *C. obtusifolia* (7.5 g), *L. chuanxiong* (6 g), *A. sinensis* (6 g), *G. uralensis* (6 g) were refluxed with water (w/v 1:8) for 1 h. The extracts collected were cool-dried by vacuum into a brown powder. Two batches of extract were prepared and the yields were ~18 %, respectively. The powder was used for in vitro and in vivo studies. An HPLC-diode array detection system (Waters) was used for fingerprint analysis. Each filtered sample (20 μL) was separated on a C18 column with a mixture of acetonitrile and 0.1 % trifluoroacetic acid in water at room temperature; a gradient elution of acetonitrile from 10–90 % was applied. Eluents were monitored by the absorbance at 254 nm.

### PSR administration

PSR dissolved in water was orally administered to rats before and after MCAO. The human-equivalent dose (HED) in rats was calculated to be 1.1 g/kg. Rats were dosed with 1.1, 2.2 and 5.5 g/kg and the dosing volume was 5.5 mL/kg. For the pre-treatment regimen, PSR was administered daily for 5 days before and 6, 24, and 48 h after MCAO onset. For the post-treatment regimen, PSR was administered 6, 24 h, and then daily after MCAO onset. Deionized water was administered as control.

### Evaluation of neurological deficit

Bederson’s neurological examination scoring system [49, 50] was used to evaluate neurological deficit. Rats were scored 0 if they had no observable deficit (normal); 1 if they failed to fully extend the left forepaw (mild deficit); 2 if they circled towards the contralateral side (moderate deficit); and 3 if they lost the ability to walk and righting reflexes (severe deficit).

### Measurement of cerebral infarction and edema extent

After the final evaluation of neurological deficit, rats were sacrificed and their brains were removed, fixed with 4 % paraformaldehyde, sectioned into seven 2-mm coronal slices, and stained with 2 % (w/v) 2,3,5-triphenyltetrazolium chloride (TTC) for 30 min at 37 °C [49]. Images of the slices were taken and analyzed using ImageJ System software (National Institutes of Health, Bethesda, MD). The infarct area in each slice was determined by subtracting the TTC-stained area in the ipsilateral (i.e., ischemic) hemisphere from that of the contralateral (i.e., non-ischemic) hemisphere and expressed as the percentage of the TTC-stained area of the contralateral hemisphere. Infarct volume was calculated on the basis of the infarct area and slice thickness. The ischemia-induced gain in hemispheric volume (i.e., edema) was calculated by subtracting the volume of the contralateral hemisphere from that of the ipsilateral hemisphere and expressed as the percentage of the volume of the contralateral hemisphere.

### Primary neuronal cultures

Embryonic cortical and hippocampal neurons express high levels of GluN2B-containing NMDA receptors and therefore were used for this study [46]. They were prepared from embryonic day 18 (E18) SD rats as described previously [51]. Cortical neurons were cultured on poly-d-lysine–coated plates and maintained in neurobasal medium supplemented with 2 % B-27 supplement, penicillin (100 units/mL), and streptomycin (100 μg/mL) at 37 °C in a humidified atmosphere with 5 % CO_2_. Hippocampal neurons were grown similarly, except the medium was supplemented with 0.5 mM L-glutamine.

### NMDA excitotoxicity assay and immunohistochemistry

Since a larger quantity of cortical neurons can be obtained from one embryo compared to hippocampal neurons, cortical neurons were used for NMDA excitotoxicity assay. Cortical neurons at 11–12 days in vitro (DIV) were pre-treated with PSR or vehicle (DMSO) for 2 h and subsequently rinsed with Locke’s solution (5 mM KCl, 128 mM NaCl, 2.7 mM CaCl_2_, 1 mM Na_2_HPO_4_, 5 mM HEPES, and 10 mM glucose) followed by a 15-min incubation with glycine-containing (10 μM) Locke’s solution. The neurons were then co-treated with PSR (or vehicle) and 20 μM NMDA dissolved in Locke’s plus glycine solution for 20 min. The neurons were then incubated with fresh growth medium. After 24 h, NMDA excitotoxicity was assessed by either the lactate dehydrogenase (LDH) release assay (Roche) or immunohistochemistry, as described previously [52]. The cell death in the presence of PSR and NMDA was quantified by normalizing the LDH release to the maximum (vehicle with NMDA, the control) and minimum (no NMDA) LDH releases. Neurons were immunostained with β-tubulin type III antibody (1:1000, Sigma) and labeled with FITC-conjugated secondary antibody. DAPI (4′, 6-diamidino-2-phenylindole) was used to stain the nuclei. Images were captured and visualized by fluorescent microscopy (Leica).

### Electrophysiology

Embryonic hippocampal neurons express larger NMDAR current compared to cortical neurons and were used for patch clamp recording of the NMDAR current. Whole-cell recording was performed at room temperature (~22 °C) as previously described [51, 53]. The pipette solution contained (in mM): 135 CsCl, 0.5 EGTA, 2 MgCl_2_, 4 Na_2_ATP, 0.4 NaGTP, and 10 HEPES (pH 7.25). The bath solution contained (in mM): 5.3 KCl, 0.4 KH_2_PO_4_, 4.2 NaHCO_3_, 138 NaCl, 0.3 Na_2_HPO_4_, and 27.8 glucose, 2.5 CaCl_2_, 0.01 glycine, 0.0005 tetrodotoxin, 0.02 bicuculline methiodide (Tocris), and 5 HEPES (pH 7.4). NMDA alone (20 μM) or with PSR were added to the bath solution. Pipettes were fire-polished, and resistance was 3–5 MΩ. Currents were recorded at −70 mV using a Multiclamp 700B amplifier and data were analyzed using pClamp 10 (Molecular Devices, USA).

### Western blotting

After treatment, cortical neurons were lysed with RIPA buffer at different time points. Western blot analysis was performed as described previously [54]. Briefly, protein samples (30 μg) were separated by SDS-PAGE and then transferred to nitrocellulose membrane, which was subsequently incubated with antibodies specific to phospho-p44/42 mitogen-activated protein kinase (p-ERK) (1:1000), p44/42 mitogen-activated protein kinase (ERK) (1:1000), phospho-cAMP response element-binding protein (p-CREB) (1:1000), or CREB (1:1000). The membranes were then washed and incubated with horseradish peroxide-conjugated secondary antibody (1:2000). Signals were detected using the SuperSignal West Pico chemiluminescent substrate kit (Pierce, Rockford, IL, USA). Band densitometry was measured and analyzed by ImageJ.

### Luciferase reporter assay

Freshly isolated cortical neurons were transiently transfected with the luciferase reporter construct (CRE-Luc) [55] using Lipofectamine 2000. Transfected neurons (9–11 DIV) were treated with PSR or vehicle (DMSO) for 6 h and then lysed with luciferase lysis buffer. Lysates were transferred onto white-bottom plates and assessed using a luciferase assay kit (Promega). Luciferase (i.e., CRE) activity was quantified using a Promega GloMax™ 96 microplate luminometer.

### Statistical analysis

Results are reported as mean ± SEM (or SD if number of experiments is 2). Data with two dependent variables (e.g. drug dose and time/coronal slice) were analyzed by two-way ANOVA followed by Bonferroni post-test; data with one dependent variable were analyzed by one-way ANOVA followed by Newman-Keuls post-test, except when stated otherwise. The level of significance was indicated by *, **, and *** which corresponds to *P* < 0.05, <0.01, and <0.0,01, respectively.

## Results

### HPLC fingerprinting of PSR extracts

**Fig. 1:**
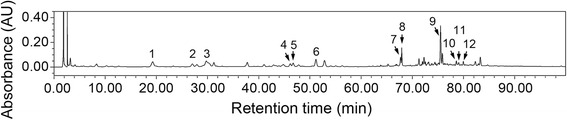
The HPLC chromatographic fingerprint of PSR. A typical chromatogram contains 12 peaks representing chemical markers of different component herbs in PSR. The 12 peaks were identified by comparing their retention times and UV spectra with those of the standards (Table 2). The presence of these 12 compounds indicates that each component herb in PSR contribute to the chemical composition of the extract

To analyze the major chemical components in the extracts and to assess the quality of extracts obtained from different batches, samples of PSR were analyzed by HPLC. A typical chromatogram of a PSR preparation is presented in Fig. 1. By comparing their retention times and UV spectra to those of the standards, 12 chromatographic peaks were identified (Table 2). These 12 compounds encompass the known chemical markers from all the seven herbs in PSR, and they contribute to the major chemical composition of the decoction. The quality of the PSR extracts was routinely monitored and only extracts containing these 12 peaks in the chromatograms were used in our experiments.

**Table 2:** Identities and abundances of the 12 marker compounds corresponding to peaks 1–12 in the chromatogram of PSR shown in Fig. 1

Peak	Marker^a^	Retention time (min)	Abundance (%)	Herbs indicated by the marker
1	Paeoniflorin	20.20	15.95 ± 0.94	*P. lactiflora*
2	Ferulic acid	28.57	0.51 ± 0.04	*A. sinensis*
				*L. chuanxiong*
3	Liquiritin	31.20	0.43 ± 0.05	*G. uralensis*
4	Ononin	45.78	0.16 ± 0.01	*A. membranaceus*
5	Lithospermic acid	48.19	0.64 ± 0.02	*S. miltiorrhiza*
6	Salvianolic acid B	52.67	1.62 ± 0.04	*S. miltiorrhiza*
7	Glycyrrhizic acid	68.14	1.41 ± 0.04	*G. uralensis*
8	Formononetin	68.45	0.53 ± 0.01	*A. membranaceus*
9	Ligustilide	75.76	0.25 ± 0.00	*A. sinensis*
				*L. chuanxiong*
10	Cryptotanshinone	78.78	0.25 ± 0.02	*S. miltiorrhiza*
11	Tanshinone I	79.39	3.81 ± 0.21	*S. miltiorrhiza*
12	Chrysophanol	80.18	0.52 ± 0.01	*C. obtusifolia*

^a^All the marker compounds of PSR except ononin have been demonstrated to be neuroprotective in rodent models of cerebral ischemia, and ononin can protect neuronal cells against glutamate-induced cytotoxicity [32, 34, 50–58]

### PSR attenuates NMDA excitotoxicity and reduces NMDA-induced current in primary cortical neurons

**Fig. 2:**
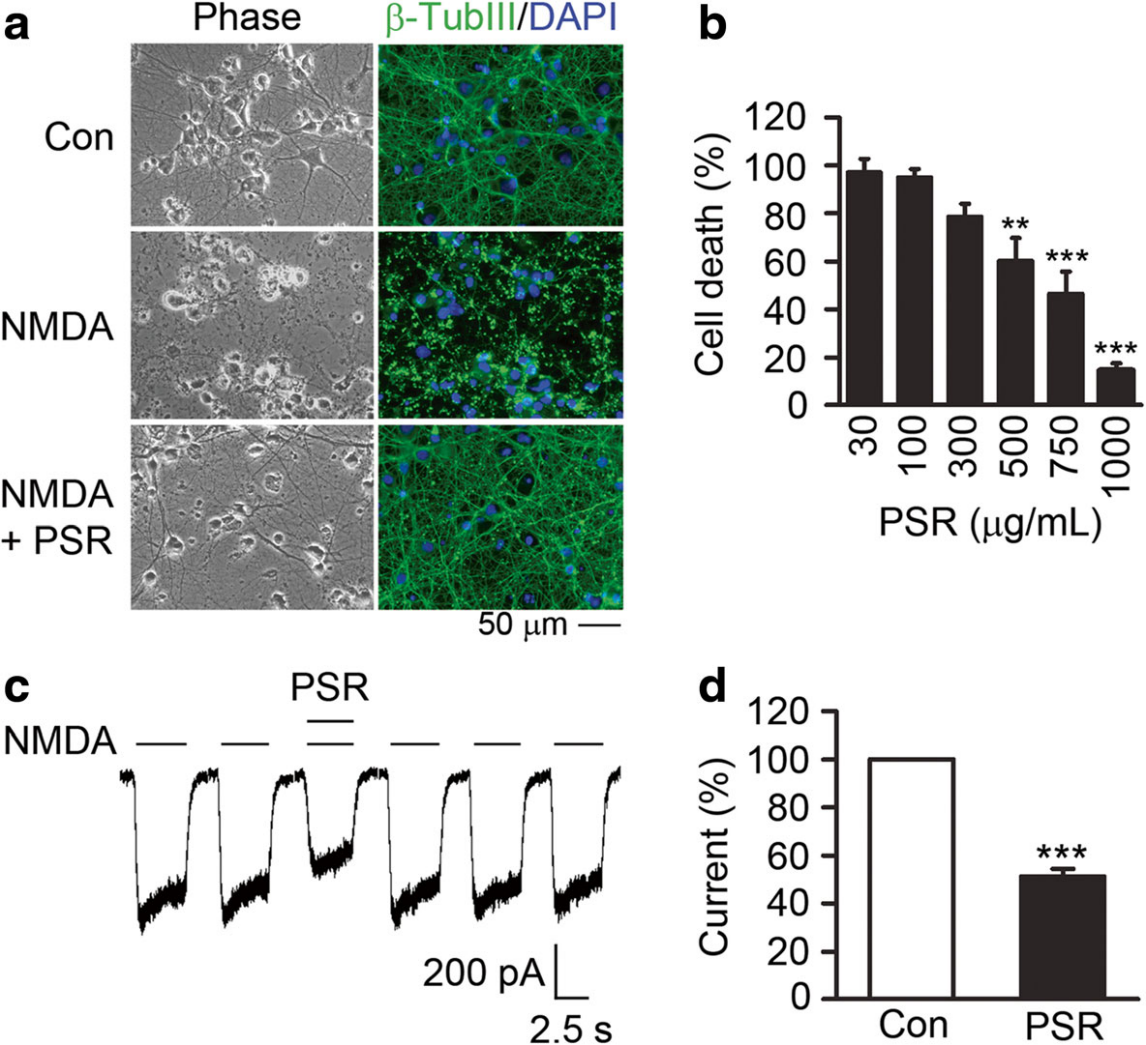
PSR protects neurons against NMDA excitotoxicity. **a** Rat cortical neurons were treated with vehicle (top panels), 20 μM NMDA (middle panels), or 20 μM NMDA plus PSR 500 μg/mL (bottom panels). Phase contrast images are shown on the left. The right panels show the overlay of the DAPI- and β-tubulin III-stained images. **b** LDH released from NMDA-challenged neurons with or without PSR treatment was measured and quantified as normalized cell death (see Methods). Four concentration-response experiments, with each concentration of PSR conducted in duplicate wells, were performed. For PSR 30, 750 and 1000 μg/mL, number of data points (n) = 2; for PSR 100, 300 and 500 μg/mL, *n* = 4. Statistical significance between PSR-treated and control groups was analyzed by one-way ANOVA followed by Newman-Keuls post-test. **c** Representative whole-cell current-response recorded from a hippocampal neuron. NMDA-induced current was reversibly inhibited by PSR 500 μg/mL. **d** Inhibitory effect of PSR 500 μg/mL on NMDA-induced current. The control was the NMDA-induced current level in the absence of PSR (set as 100 %). Statistical significance was analyzed by paired t-test (*n* = 4)

All the signature compounds of PSR, except ononin (Table 2), have been demonstrated to be neuroprotective in rodent models of cerebral ischemia, and ononin can protect neuronal cells against glutamate-induced cytotoxicity [32, 34, 50–58]. Therefore, it is likely that PSR extract prepared from our formula can exert neuroprotection. To investigate whether PSR extract is neuroprotective in vitro, we employed primary cortical neurons that have been insulted with NMDA to induce excitotoxicity. E18 cortical neurons were treated with PSR extract for 2 h followed by a 20-min co-incubation with a toxic dose of NMDA (20 μM). Immunostaining was performed 24 h after NMDA challenge. DAPI and β-tubulin type III staining were used to indicate nuclei and neurite networks in neuronal cultures, respectively. Cortical neurons insulted by NMDA exhibited shrunken cell bodies with sparse neurites (Fig. 2a). PSR 500 μg/mL treatment largely restored the intact neurite network in NMDA-challenged neurons (Fig. 2a). When cell death was quantified by LDH assay, a dose-dependent protective effect of PSR on NMDA-treated cells was revealed. PSR 500 to 1000 μg/mL treatment significantly reduced NMDA-induced cell death in primary cortical neurons (Fig. 2b). These findings demonstrate that PSR effectively protects cortical neurons against NMDA-induced excitotoxicity.

The calcium current induced by NMDA in hippocampal neurons contributes to its excitotoxicity [56]. To determine if PSR affects NMDA-evoked current, whole-cell patch-clamp recording was conducted on hippocampal neurons. PSR and NMDA co-administration resulted in reduced current (Fig. 2c). PSR 500 μg/mL significantly reduced the mean steady state currents by 49 ± 3 % compared to the control (Fig. 2d). A 5 s wash-out restored the NMDA-induced current to the control level, indicating that PSR reversibly inhibits NMDAR current with a relative fast off-rate.

### PSR activates ERK/CREB signaling in primary cortical neurons

**Fig. 3:**
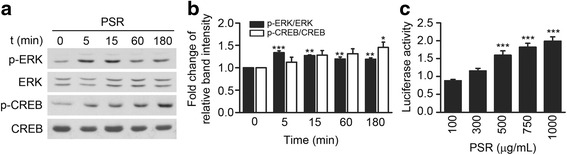
Activation of ERK and CREB by PSR. **a** Western blots and quantitative densitometry (**b**) showing the effect of 0–180 min of PSR 500 μg/mL treatment on ERK and CREB phosphorylation. Band intensities in western blots (*n* = 2) were quantified using ImageJ. The band intensity of the phosphorylated ERK/CREB protein is first normalized to that of the corresponding total ERK (or CREB) protein, and then the value of the normalized band intensity of the phosphorylated protein at time 0 was set as 1. Statistical significance in (**b**) was performed by one-way ANOVA followed by Newman-Keuls post-test to compare the band intensity at each time point to that of time 0. **c** Effect of 6 h-treatment of PSR on the luciferase activity of neurons transfected with the CRE-Luc construct. Seven concentration-response experiments, with each concentration of PSR conducted in duplicate wells, were performed. For PSR 100 to 1000 μg/mL, *n* = 5, 6, 7, 7, 4, respectively. Statistical significance between PSR-treated and control groups was analyzed by one-way ANOVA followed by Newman-Keuls post-test

ERK activation has been implicated in protecting neurons against apoptosis [57, 58]. CREB, a transcription factor involved in neuroprotection, learning, and memory, is activated downstream of ERK [59, 60]. To determine whether PSR can activate ERK and CREB, primary cortical neurons were treated with PSR 500 μg/mL and the phosphorylation levels of ERK and CREB were investigated. Western blotting revealed that ERK phosphorylation had already maximally increased after 5 min of treatment and was maintained at this high level for up to 15 min of treatment, decreasing gradually thereafter to a level still slightly higher than that of the control (band intensities were compared at 0 and 180 min of treatment; Fig. 3a, b). On the other hand, a persistent increase in CREB phosphorylation was observed, and at 180 min of treatment, the increase in CREB phosphorylation was significant. To verify the activation of CREB, the effect of PSR on CRE-promoter was examined in primary cortical neurons transiently expressing a CRE-driven luciferase reporter. Compared to the control, incubation with PSR 500–1000 μg/mL significantly activated CRE promoter, as indicated by the increase in luciferase activity (Fig. 3b). Taken together, these results suggest that PSR may enhance the survival of neurons by potentiating the pro-survival ERK and CREB signaling.

### PSR pre-treatment reduces functional deficit and ischemic injury in MCAO rats

**Fig. 4:**
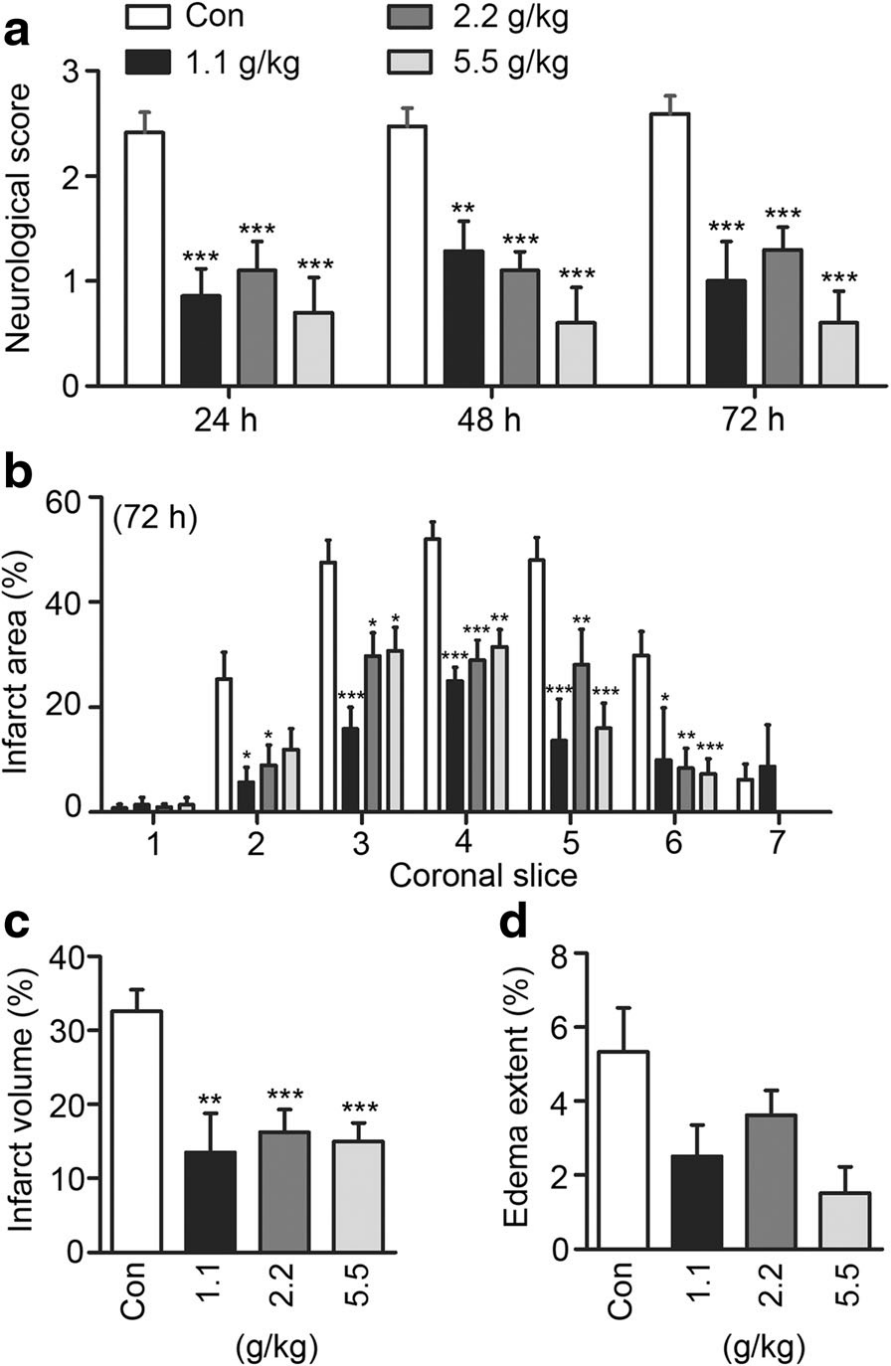
PSR pre-treatment reduces neurological deficit and cerebral infarct in MCAO rats. **a** Rats were pre-treated with vehicle (con) or different doses of PSR, and neurological scores were determined 24, 48, and 72 h after MCAO. Infarct area (**b**), infarct volume (**c**), and edema content (**d**) were measured 72 h after MCAO. For (**a**-**c**), the numbers of animals in the control, PSR 1.1, 2.2 and 5.5 g/kg groups were 17, 7, 10 and 10, respectively. For (D), n for control = 14. In (**a**, **b**), statistical significance between PSR-treated and control groups was analyzed by two-way ANOVA followed by Bonferroni post-test. For infarct area, the *P*-values of the differences between treatment groups and the corresponding controls of the same coronal slices are shown. In (**c**, **d**), statistical significance between PSR-treated and control groups was analyzed by one-way ANOVA followed by Newman-Keuls post-test. The key pattern shown in (**a**) applies to all other panels in this figure as well as in Fig. 5

Given the in vitro data showing PSR could activate pro-survival ERK and CREB signaling, inhibit NMDAR current and counteract NMDA-induced neuronal excitotoxicity, we explored whether PSR is neuroprotective against ischemic brain injury using the rat transient MCAO model (i.e., 2 h occlusion followed by reperfusion) [50]. We began by examining if PSR could reduce acute ischemic injury by using a pre-treatment regimen. MCAO strongly affected neural behavior as indicated by the neurological scores of the vehicle control groups measured at 24, 48 and 72 h after MCAO (score >2; Fig. 4a). Significant improvements (i.e., decreases) in neurological scores were observed in rats treated with PSR at all dosages tested at 24 h and the improvements were maintained at 48 and 72 h. The cerebral infarct area and volume were also reduced in PSR-treated rats (Fig. 4b, c and Additional file 1: Figure S1 in supplemental information). The extent of edema was reduced in PSR-treated rats, although the difference was not statistically significant when compared to the control (Fig. 4d). These data indicate that pre-treating rats with PSR can effectively reduce the functional deficit and cerebral infarct induced by MCAO. The physiological parameters of the rats were closely monitored throughout the experiments, and no differences were found after PSR administration with respect to mean pH, PaCO_2_, PaO_2_, [HCO_3_^−^], or [Hb] (Additional file 1: Table S1).

### PSR post-treatment attenuates functional deficit and ischemic injury

**Fig. 5:**
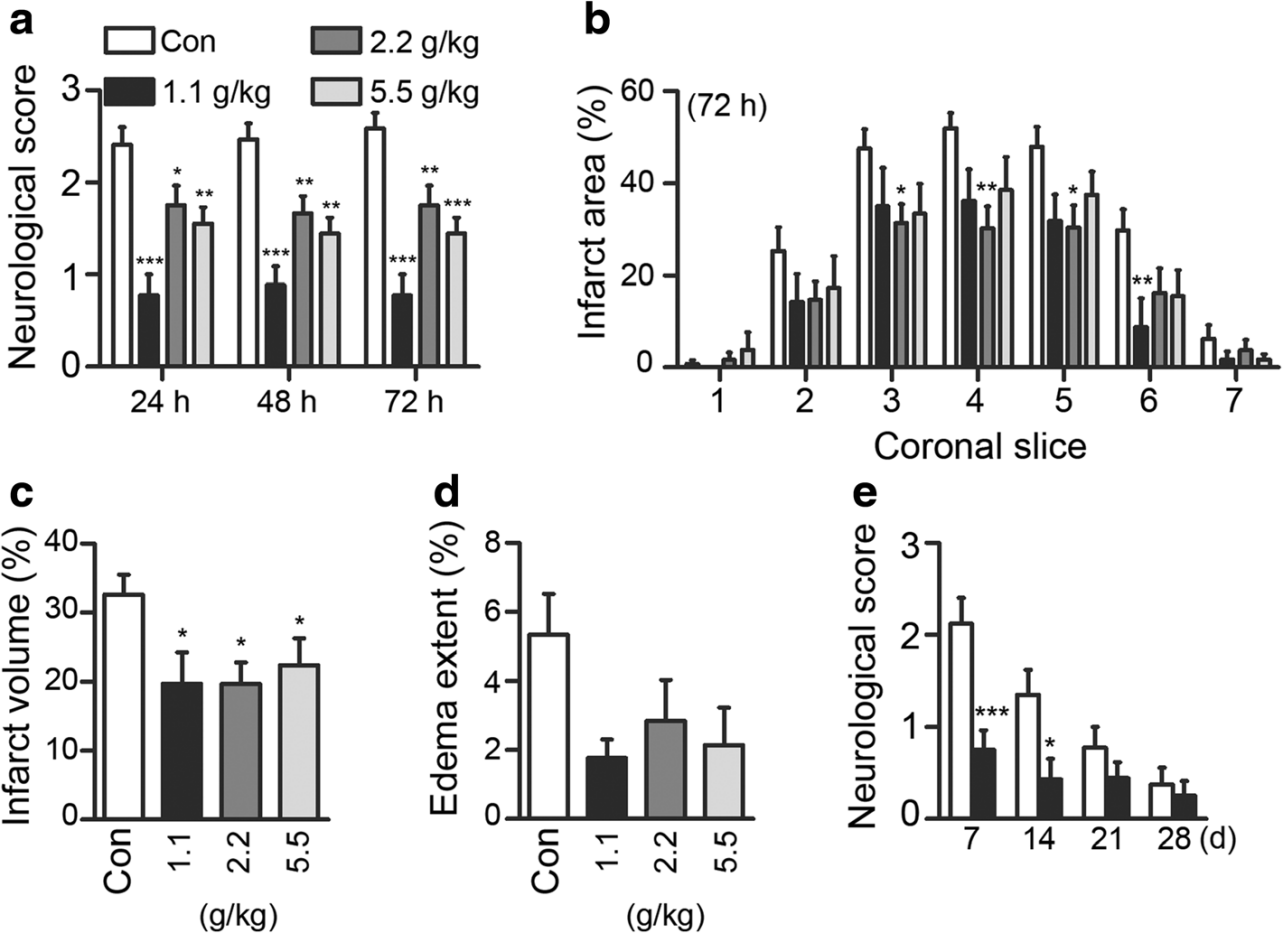
PSR post-treatment reduces neurological deficit and cerebral infarct in MCAO rats. **a** Rats were post-treated with vehicle (con) or different doses of PSR, and neurological scores were determined 24, 48, and 72 h after MCAO. Infarct area (**b**), infarct volume (**c**), edema content (**d**) were measured 72 h after MCAO. **e** Adult rats were post-treated with vehicle (con) or PSR 1.1 g/kg for up to 28 d after MCAO. Neurological scores measured at 7, 14, 21, and 28 d after MCAO are shown. For (**a**-**c**), the numbers of animals in the control, PSR 1.1, 2.2 and 5.5 g/kg groups were 17, 9, 12 and 9, respectively. For (**d**), n for control = 13. For (**e**), the numbers of animals for control and PSR 1.1 g/kg groups at day 7, 14, 21 and 28 were 17, 17, 9, 8 and 16, 14, 9, 8, respectively. In (**a, b**) and (**e**), statistical significance between PSR-treated and control groups was analyzed by two-way ANOVA followed by Bonferroni post-test, respectively. In (**c**-**d**), statistical significance between PSR-treated and control groups was analyzed by one-way ANOVA followed by Newman-Keuls post-test

In order to investigate the therapeutic efficacy of PSR against acute stroke in a more clinically relevant setting, PSR was administered 6, 24 and 48 h after MCAO onset (post-treatment regimen). Rats post-treated with PSR at all given doses exhibited significant improvements in neurological scores measured 24, 48, and 72 h after MCAO (Fig. 5a); the greatest improvement was achieved with PSR 1.1 g/kg. Consistent with the improvement in neural behavior, there were overall reductions in infarct area and volume in MCAO rats post-treated with PSR (Fig. 5b, c and Additional file 1: Figure S2). The extent of edema was reduced in PSR-treated rats, although not statistically significant (Fig. 5d). As neurological impairment is a major long-term complication in stroke patients, we evaluated if PSR can attenuate the devastating long-term consequences of behavioral impairment in MCAO rats. In this experiment, rats were post-treated with PSR 1.1 g/kg for up to 4 weeks and the subsequent neurological functions were examined once weekly. Compared to the control rats, PSR significantly improved the neurological scores of rats at 7 and 14 days after MCAO (Fig. 5e). Taken together, these data demonstrate that PSR post-treatment can effectively alleviate ischemic injury and behavioral deficit in rats after cerebral ischemia.

## Discussion

PSR is a novel neuroprotective formula designed for stroke treatment. Its constituent herbs have all been demonstrated with prominent neuroprotective properties. The further development of PSR for stroke treatment relies on both preclinical and clinical studies. The work reported here is a pioneer preclinical study of PSR demonstrating its neuroprotective efficacy. We first demonstrated that PSR extract could prominently attenuate cell death using cultured NMDA challenged-neurons. This in vitro finding validate the neuroprotective character of the selected herbal components of PSR. Furthermore, it indicate that the net activity of all the neuroprotective compounds within the PSR extract persists despite possible neutralizing interactions that can occur with the many other molecules presence in the extract. Support by the in vitro data, PSR was tested in vivo using MCAO rats, and demonstrated to be effective in reducing cerebral injury and improving neural behavior. Further in vitro studies on the effects of PSR on NMDAR currents and ERK and CREB activation shed insights into its mechanism of action.

Our in vivo study demonstrates that PSR pre- and even post-treatments can effectively decrease ischemic infarct size and reduce functional neurological impairment in MCAO rats. The effectiveness of pre-treatment suggests that PSR may be developed for the prophylactic management of patients with a high risk of stroke recurrence [61]. Two aspects of the present post-treatment data are particularly outstanding. First, PSR acts quickly to mediate neuroprotection and elicits sustained improvement in neurological function in MCAO rats. Improvements in neurological scores were observed as early as 18 h after the first dose of PSR and extended up to day 14. Second, the therapeutic time window of PSR in rats is at least 6 h after the onset of MCAO, which is longer than many other tested compounds/drugs in similar animal models. As patients with stroke onset longer than 3 h before therapeutic intervention can be practically applied is common, the 6 h therapeutic time window of PSR in MCAO rats increases the chance of PSR to be successfully translated to human stroke patients [14, 62].

Our in vitro finding that PSR can reversibly inhibit NMDA-induced currents with a fast off-rate provides one possible mechanism underlying the neuroprotective effect of PSR. A fast off-rate is a desirable pharmacological property of an NMDAR current inhibitor critical for safety [11, 53], and the fast off-rate of PSR is consistent with the long safety history of the herbal components of PSR to treat humans. In contrast to memantine, another NMDAR blocker with fast off-rate failed to protect against ischemic impairment in rat when administered 1 h after the onset of MCAO [47], the superior therapeutic effect of PSR when administered 6 h after ischemia suggests that mechanisms other than NMDAR current inhibition is involved in mediating its potent neuroprotective effect.

Increasing evidence shows that single-targeted drug candidates are ineffective for stroke treatment [63]. As TCM formulas contain multiple herbs designed and assumed to act synergistically [19–21], their pharmacological actions are usually multi-targeted. PSR indeed has multiple targets – in addition to inhibiting NMDAR-mediated current, PSR can activate ERK and CREB, which may potentiate the expression of CREB-mediated genes such as growth factors and anti-apoptotic proteins to exert neuroprotective effects [64–67]. Two of the constituent herbs, *A. sinensis* and *L. chuanxiong*, have been demonstrated or implicated in activating CREB – pretreatment of cortical neuron culture with extract of *A. sinensis* prevented the reduction of phospho-CREB induced by amyloid beta peptide, and extract of *L. chuanxiong* induced CREB phosphorylation in PC12 cells [68, 69]. However, to the best of our knowledge, no decoction has been reported to enhance ERK and CREB signaling in cultured primary neurons. Only until recently, a decoction of thirteen herbs to treat memory impairment of schizophrenia patient was shown to activate ERK and CREB in hippocampi of treated animals [70]. Moreover, PSR likely acts on other targets in addition to NMDAR and ERK/CREB, as all its seven constituent herbs except *C. obtusifolia* have been implicated to have both anti-oxidative and anti-inflammatory effects, while *C. obtusifolia* has been implicated to have only anti-inflammatory effect [71–77]. In future experiments, it will be important to demonstrate the anti-oxidative and anti-inflammatory effects of PSR extract, and explore other plausible mechanisms that can contribute to its neuroprotective effect.

There are two possible directions regarding the further development of PSR. First, the PSR extract, although a complex molecular mixture, can be further investigated and developed for stroke treatment. Such effort requires the preparation of a quality-controlled extract from the seven constituent herbs, which in turn relies on standardized good practice to ensure the use of high quality herbs, a consistent procedure for extract preparation and chemical profiling to validate the quality the extract [78, 79]. Alternatively, the extract can be simplified by systematically purifying molecular fractions from the seven herbs, identifying the active molecules, and mixing the active molecules in optimized molar ratio to generate a cocktail which can achieve the best neuroprotective effects. The optimized molar ratio of the active molecules can be derived by using computational algorithm coupled to bioassays [80].

## Conclusions

Extract of PSR, a novel Chinese herbal formula, is neuroprotective in cultured neurons and is effective in reducing ischemic injury in MCAO rats in both the pre- and post-treatment regimens, and the underlying neuroprotective mechanisms may involve inhibition of NMDA receptor current and activation of ERK and CREB. As neuroprotection strategies continue to show promise in the treatment of ischemic and hemorrhagic types of strokes, our study provides the scientific basis and critical preclinical data for further development of PSR for stroke treatment.

## Additional files

Additional file 1: Table S1. Assessments of physiological parameters before, during and after ischemia (MCAO) in control and PSR pre-treated rats. **Figure S1.** Representative TTC-stained brain slices for sham and MCAO rats, with the latter animals divided into 4 groups that were pre-treated with vehicle, 1.1 g/kg, 2.2 g/kg or 5.5 g/kg PSR. **Figure S2.** Representative TTC-stained brain slices for sham and MCAO rats, with the latter animals divided into 4 groups that were post-treated with vehicle, 1.1 g/kg, 2.2 g/kg or 5.5 g/kg PSR. (DOCX 3065 kb)
Additional file 2: An excel file containing all the data used for plotting the bar charts. (XLSX 14 kb)

## Abbreviations

ANOVA: Analysis of variance
ATP: Adenosine 5′-triphosphate
CREB: cAMP response element binding protein
DAPI: 4′,6-diamidino-2-phenylindole
DMSO: Dimethyl sulfoxide
EGTA: Ethylene glycol-bis (2-aminoethylether)-N,N,N′,N′-tetraacetic acid
ERK: Extracellular signal–regulated kinase
FDA: Food and drug administration
GABA: Gamma-aminobutyric acid
GTP: Guanosine-5′-triphosphate
HEPES: 4-(2-Hydroxyethyl) piperazine-1-ethanesulfonic acid
HPLC: High performance liquid chromatography
LDH: Lactate dehydrogenase
MCAO: Middle cerebral artery occlusion
NMDAR: N-methyl-D-aspartate receptor
PSD95: Postsynaptic density protein 95
SD: Standard error
SDS-PAGE: Sodium dodecyl sulfate polyacrylamide gel electrophoresis
SEM: Standard error of the mean
