# Supplementary material for: Neuroprotective effect of a novel Chinese herbal decoction on cultured neurons and cerebral ischemic rats
Source: BMC Complement Altern Med. 2016 Nov 4;16:437. doi: 10.1186/s12906-016-1417-1 (PMC5097373; doi:10.1186/s12906-016-1417-1)

**Supplemental information**

**Table S1**

Assessments of physiological parameters before, during and after ischemia (MCAO) in control and PSR pre-treated rats

| Parameter | Group | Before ischemia | During ischemia | After ischemia |
| --- | --- | --- | --- | --- |
|  |  |  |  |  |
| pH | vehicle | 7.26 ± 0.02 | 7.23 ± 0.03 | 7.22 ± 0.05 |
|  | PSR 0.62 g/kg | 7.26 ± 0.04 | 7.23 ± 0.04 | 7.21 ± 0.02 |
|  | PSR 1.24 g/kg | 7.25 ± 0.01 | 7.24 ± 0.03 | 7.20 ± 0.03 |
|  | PSR 3.1 g/kg | 7.27 ± 0.01 | 7.22 ± 0.01 | 7.21 ± 0.01 |
| PaCO_2_^*^ | vehicle | 53.20 ± 1.72 | 53.85 ± 3.60 | 51.13 ± 5.32 |
| (mmHg) | PSR 0.62 g/kg | 53.43 ± 3.46 | 57.37 ± 2.99 | 55.13 ± 4.87 |
|  | PSR 1.24 g/kg | 53.83 ± 3.40 | 56.40 ± 4.60 | 56.87 ± 1.39 |
|  | PSR 3.1 g/kg | 55.40 ± 4.20 | 58.65 ± 0.95 | 54.70 ± 1.65 |
| PaO_2_ | vehicle | 95.75 ± 2.29 | 107.00 ± 6.65 | 124.75 ± 11.00 |
| (mmHg) | PSR 0.62 g/kg | 101.67 ± 6.17 | 116.00 ± 10.44 | 127.33 ± 8.97 |
|  | PSR 1.24 g/kg | 98.67 ± 12.12 | 104.33 ± 19.67 | 136.67 ± 10.17 |
|  | PSR 3.1 g/kg | 98.50 ± 5.50 | 115.00 ± 10.00 | 130.00 ± 9.00 |
| HCO_3_^-^ | vehicle | 23.63 ± 0.49 | 22.48 ± 0.81 | 20.60 ± 0.64 |
| (mmol/L) | PSR 0.62 g/kg | 23.63 ± 0.78 | 24.17 ± 0.87 | 24.70 ± 0.38 |
|  | PSR 1.24 g/kg | 23.83 ± 1.60 | 23.97 ± 1.52 | 22.20 ± 1.30 |
|  | PSR 3.1 g/kg | 25.40 ± 1.30 | 24.40 ± 0.40 | 22.05 ± 0.95 |
| Hb | vehicle | 17.93 ± 0.47 | 17.15 ± 0.68 | 18.20 ± 0.96 |
| (g/dL) | PSR 0.62 g/kg | 17.90 ± 0.32 | 18.47 ± 0.29 | 18.83 ± 0.65 |
|  | PSR 1.24 g/kg | 18.37 ± 0.38 | 18.47 ± 0.62 | 19.70 ± 0.00 |
|  | PSR 3.1 g/kg | 17.85 ± 1.15 | 17.35 ± 0.35 | 17.85 ± 0.85 |

^*^PaCO_2_, PaO_2_ and Hb refer to the partial pressure of carbon dioxide and oxygen, and haemoglobin, respectively.

**Figure S1**

Representative TTC-stained brain slices for sham and MCAO rats, with the latter animals divided into 4 groups that were pre-treated with vehicle, 1.1 g/kg, 2.2 g/kg or 5.5 g/kg PSR.


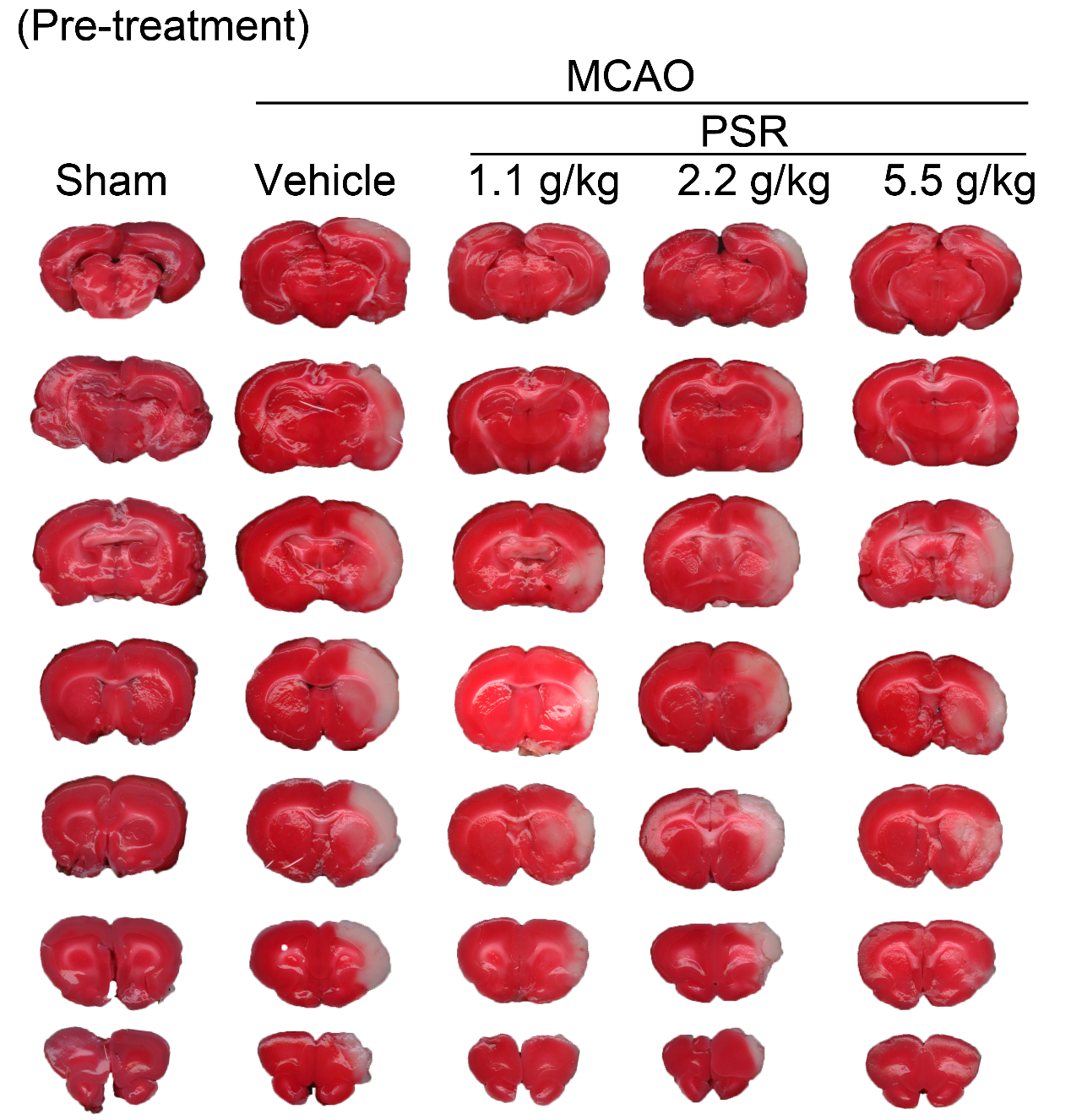


**Figure S2**

Representative TTC-stained brain slices for sham and MCAO rats, with the latter animals divided into 4 groups that were post-treated with vehicle, 1.1 g/kg, 2.2 g/kg or 5.5 g/kg PSR.


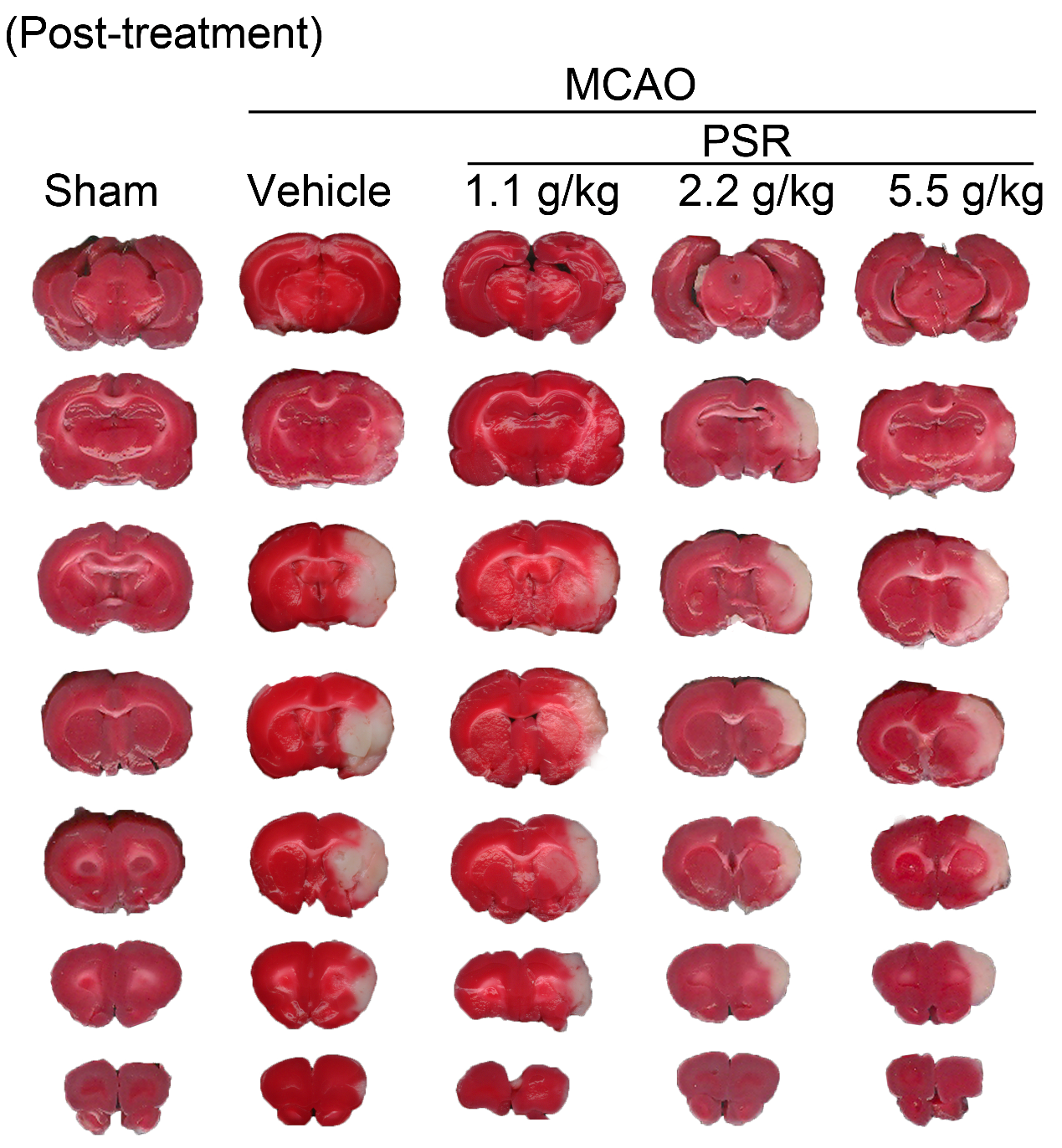

Supplement: Additional file 1: Table S1. — Assessments of physiological parameters before, during and after ischemia (MCAO) in control and PSR pre-treated rats. Figure S1. Representative TTC-stained brain slices for sham and MCAO rats, with the latter animals divided into 4 groups that were pre-treated with vehicle, 1.1 g/kg, 2.2 g/kg or 5.5 g/kg PSR. Figure S2. Representative TTC-stained brain slices for sham and MCAO rats, with the latter animals divided into 4 groups that were post-treated with vehicle, 1.1 g/kg, 2.2 g/kg or 5.5 g/kg PSR. (DOCX 3065 kb) [file 12906_2016_1417_MOESM1_ESM.docx]
